# An absolutely dated mid-Holocene English yew chronology offers new opportunities for archaeological and palaeoenvironmental research

**DOI:** 10.1177/09596836251407634

**Published:** 2026-02-03

**Authors:** Tatiana Bebchuk, Darren Davies, Neil J Loader, Otmar Urban, Tito Arosio, Lukas Wacker, Natálie Pernicová, Josef Čáslavský, Miroslav Trnka, Alexander V Kirdyanov, David Brown, Jan Esper, Clive Oppenheimer, Ulf Büntgen

**Affiliations:** 1 Department of Geography, University of Cambridge, UK; 2 Department of Geography, Swansea University, UK; 3 Global Change Research Institute, Czech Academy of Sciences, Czech Republic; 4 Swiss Federal Institute for Forest, Snow and Landscape Research WSL, Switzerland; 5 Laboratory of Ion Beam Physics, ETHZ, Switzerland; 6 Department of Agrosystems and Bioclimatology, Mendel University in Brno, Czech Republic; 7 Siberian Federal University, Russia; 8 Sukachev Institute of Forest SB RAS, Federal Research Center ‘Krasnoyarsk Science Center SB RAS’, Russia; 9 School of Natural and Built Environment, The Queen’s University, UK; 10 Department of Geography, Johannes Gutenberg University, Germany; 11 Department of Geography, Masaryk University, Czech Republic

**Keywords:** archaeology, cross-dating, dendrochronology, multi-proxy, palaeoclimatology, radiocarbon dating, stable isotopes, tree rings

## Abstract

Research on Holocene climate variability and human history greatly benefits from annually resolved and absolutely dated tree-ring chronologies. The quality and quantity of such records, however, decline back in time, with little tree-ring evidence available for the Early and Middle Holocene. Here, we present a tree-ring width (TRW) chronology from 100 subfossil yews (*Taxus baccata* L.) excavated from peat-rich soils in the Fenland region of eastern England. To precisely date the record, we measured stable oxygen (δ^18^O) isotopic ratios of over 1500 tree rings from a subset of 12 disc samples and used an absolutely dated oak (*Quercus* spp.) δ^18^O chronology from the same region for cross-dating. Statistically significant isotopic agreement between the two species precisely dates the yew TRW chronology from 2668 to 2213 years BCE (*r* = 0.4, *t*-value = 7.9, probability of error > 10^6^, Isolation Factor > 10^3^). This 456-year period in the mid-Holocene marks the Neolithic-to-Bronze Age transition, coincides with the spread of the Bell Beaker culture across the British Isles, and precedes the still debated 4.2 ka climate anomaly. Emphasizing the advantages of tree-ring stable isotopes, our absolutely dated yew record offers new opportunities for archaeological interpretations and palaeoclimatological reconstructions in eastern England and beyond. We further expect our results to help dating the Icelandic eruptions of Katla and Hekla 4 and refining the next radiocarbon calibration curve.

## Introduction

Due to their annual resolution and absolute dating, tree-ring chronologies play an essential role in archaeological, climatic, and environmental research (Cook and Kairiukstis, 2013; Fritts, 1976). Further to dating relict wood remains (Bannister and Robinson, 1975; Tegel et al., 2022), dendro data are extensively used for reconstructing temperature and hydroclimate variability over centuries to millennia (e.g. Esper et al., 2016; Ljungqvist et al., 2020). Tree-ring records may also contain precise signatures of post-volcanic summer cooling (e.g. D’Arrigo et al., 2001; Esper et al., 2013), contribute to the interpretation of historical events (e.g. Büntgen et al., 2016; Oppenheimer et al., 2018), and refine the international radiocarbon (^14^C) calibration curves (IntCal; Reimer et al., 2020; Reinig et al., 2020). The quantity and quality of relict wood from different sources, however, decrease back in time, and limited tree-ring evidence is available for the early- and mid-Holocene (Bebchuk et al., 2024; Büntgen and Esper, 2025).

**Figure 1.**
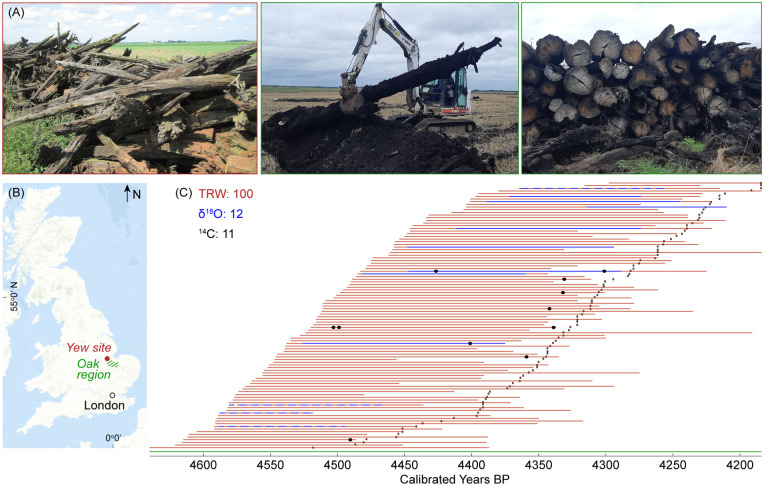
Samples characteristics. (a) Photos of sampling sites with subfossil yews (left) and oaks (middle and right). (b) Sampling locations of subfossil yew (red) and oak (green) wood. (c) Temporal distribution of 100 yew tree-ring width (TRW, red bars) series provisionally radiocarbon (^14^C) dated and sorted by their first ring. Small dots indicate sample distribution when sorted by end dates. The 12 samples used for stable oxygen isotope (δ^18^O) analysis are shown in blue, of which eight passed the ISODATE statistical thresholds (solid line) and four did not pass (dashed line). Black dots mark the samples used for ^14^C analysis. Data are in calibrated years Before Present (BP) at an uncertainty of 7 years at the 95.4% probability. The green bar shows the span of the reference oak δ^18^O chronology. *Note*. Please refer to the online version of the article to view this figure in color.

The Fenland region in eastern England comprises a vast, yet rapidly disappearing archive of subfossil wood (Figure 1; Bebchuk and Büntgen, 2025). Since the 1630s, this peat-rich flat lowland spreading at sea-level from Cambridge in the south to Lincoln in the north and from Peterborough in the west to the Wash Bay in the east, has been extensively drained for agricultural use (Darby, 1940). As peat thickness was reducing due to intensive farming, wind erosion and decomposition of organic materials, countless oak, yew, pine, willow, elm, birch, and ash trees came to the surface and have been excavated and subsequently burnt by generations of farmers (Astbury, 1958; Darby, 1983; Ennion, 1951; Godwin, 1978; Pryme, 1701; Pryor, 2019; Skertchly, 1877). Although the antiquity of trees was long recognized, their age remained unknown. English antiquary Abraham de la Pryme was the first to systematically describe these ‘subterraneous trees’ in 1701, arguing in his letter to the *Philosophical Transactions* that ‘Romans were the destroyers of all those great Woods and Forests’ (Pryme, 1701). After a gap of almost two centuries, English geologist Sidney B. J. Skertchly mapped the stratigraphy of these buried trees and hypothesized that some of them ‘may date back 70,000 years’ (Miller and Skertchly, 1878; Skertchly, 1877). It was only in the 1980s that dendrochronology was applied to contemporaneously unearthed oak trunks, so-called ‘bog oaks’, resulting in a 1,500-year-long TRW chronology for the mid-Holocene (Baillie and Brown, 1988). This record was absolutely dated against two continuous oak chronologies from Ireland and Germany (Brown et al., 1986; Pilcher et al., 1984). The recent academic discovery of hundreds of exceptionally well-preserved yew trunks has given new momentum to Fenland dendrochronology (Bebchuk et al., 2024, 2025). Despite its large sample size and close linkages to changes in sea level, the mid-Holocene yew chronology from eastern England is still floating and only absolute calendar dating will release its full potential for archaeological and paleoenvironmental research.

Calendar dates of wood samples are usually established via cross-dating – a fundamental technique in dendrochronology that associates ring width patterns with absolute calendar dates (Douglass, 1941; Wigley et al., 1987). While the method works reliably at climatically extreme sites, it may fail where tree growth is not sufficiently stressed by a single dominant climate factor. The sparse distribution of subfossil wood finds worldwide, along with the limited number of contributing tree species further constrains the potential to develop absolutely dated tree-ring records over several millennia and from different regions and species (Tegel et al., 2022). To varying extents, most tree-ring chronologies for the early- and mid-Holocene therefore rely on provisional radiocarbon dating, which has resulted in important, yet floating tree-ring chronologies being developed for England (Batchelor et al., 2020), Greece (Christopoulou et al., 2024), Albania and North Macedonia (Hafner et al., 2021), Turkey (Pearson et al., 2020), Egypt (Kuniholm et al., 2014), and China (Shi et al., 2025), for example.

Tree-ring stable oxygen isotopes (δ^18^O) have been recently employed as an alternative dating tool (e.g. McCarroll et al., 2019; Nakatsuka et al., 2020; Römer et al., 2023). The δ^18^O in tree-ring cellulose is captured in wood without the need for plants to be climatically stressed as it is primarily controlled by the isotopic composition of source water and evaporative enrichment during transpiration (McCarroll and Loader, 2004; Siegwolf et al., 2022). As a result, a common δ^18^O signal is usually recorded even under favourable growth conditions at non-extreme sites with mild climates (Hartl-Meier et al., 2015; Loader et al., 2008). Another advantage of δ^18^O over traditional TRW cross-dating is that a relatively small sample replication and short measurement series can be sufficient to develop a reliable δ^18^O chronology (Loader et al., 2019). These benefits have facilitated cross-dating of δ^18^O records for different species (Loader et al., 2021) and broad geographical regions (Haneca et al., 2025; Shi et al., 2025), with promising applications for archaeological dating (Nayling et al., 2024a, 2024b; Sano et al., 2022).

Here, we present a TRW chronology from a 100 subfossil yew (*Taxus baccata* L.) trees from eastern England and analyse δ^18^O in the annual growth rings of selected samples to enable precise dating against an absolutely dated oak (*Quercus* spp.) δ^18^O chronology from the same region. We discuss the implications of our results for archaeological and environmental research and further demonstrate the potential of our dataset to contribute to the next IntCal product.

## Data and methods

Between 2020 and 2024, we collected cross-sectional discs from over 400 subfossil yew trees in the Fenland region of eastern England (Figure 1a and b) and measured TRW along 2−5 radii per sample (for details, see Bebchuk et al., 2024). Visual cross-dating of all TRW measurements and statistical verification were performed using the TSAP-Win and COFECHA software, respectively (Holmes, 1983; Rinn, 1996). To provisionally date the yew record, 11 blocks of 10−20 tree rings from 8 samples were selected for ^14^C analysis. The samples were processed via the base-acid-base-acid method followed by bleaching and graphitization, using a high precision MICADAS facility at ETH in Zurich, Switzerland (Figure 1c; Němec et al., 2010; Sookdeo et al., 2020; Wacker et al., 2010). All ^14^C dates were wiggle-matched (Bronk Ramsey et al., 2001) and statistically resolved against IntCal20 (Reimer et al., 2020) using the OxCal v.4.4 software (Bronk Ramsey, 1995, 2021). The resulting 456-year-long TRW chronology comprises 100 yew samples (Supplemental Table S1) and spans from 4634 to 4179 ±7 calibrated years BP at the 95.4% probability (Figure 1c). Since no yew TRW chronology extends continuously from the present back into the mid-Holocene, we compared our record against an absolutely dated oak TRW chronology from the same region (Figure 1a), which was developed at Queen’s University in Belfast (QUB) in the 1980s (Baillie and Brown, 1988). We also compared our floating yew TRW chronology against English pine and German oak TRW chronologies (Billamboz, 2002, 2003; Boswijk, 1998). However, no statistically significant match was found with any of the existing chronologies.

To overcome these dating constraints, we analysed δ^18^O values from the tree rings of 12 yew samples (Figure 1c). Annual rings, including both early- and latewood, were split under a stereo microscope, with alpha-cellulose extracted following the modified Jayme-Wise isolation method (Boettger et al., 2007). Approximately 1 mg of the material was pyrolysed to carbon monoxide (CO) at 1450°C using a varioPYROcube elemental analyser (Elementar Analysensysteme, Germany), and the stable oxygen isotopes in the CO gas were measured using an ISOPRIME100 continuous-flow Isotope Ratio Mass Spectrometer (IRMS; IsoPrime, Manchester, UK) at the Global Change Research Institute in Brno, Czech Republic. Prior to each set of analyses, the IRMS was tuned and tested for signal stability (standard deviation ⩽ 0.04‰ over 10 pulses of reference gas) and linearity (⩽0.03‰/nA) across the expected ion current range obtained from the measurements of the test samples. Precision was maintained with standard deviations ⩽0.10‰ based on 5 consecutive measurements of the same alpha-cellulose sample. Isotopic values were calibrated against certified reference materials from the International Atomic Energy Agency (IAEA, Vienna, Austria). The δ^18^O values were referenced to benzoic acids (IAEA-601 and IAEA-602), and are reported in permil (‰) relative to Vienna Standard Mean Ocean Water (VSMOW; Coplen, 1995).

The individual yew δ^18^O series were cross-dated using the novel ISODATE software package (Davies et al., 2025). After indexation with a 9-year rectangular filter by subtraction, the series were first compared against each other and then against a reference oak δ^18^O chronology that is currently under development at Swansea University, UK.

To date, the reference oak δ^18^O chronology is anchored using ring width dendrochronology and has a near continuous coverage between 2982 and 1954 BCE (datum considers the year zero; Büntgen and Oppenheimer, 2020). It consists of 15 oak samples, out of which 11 form part of the absolutely dated TRW chronology developed at QUB by Baillie and Brown (1988), and the other four samples were recently included to the chronology to increase its sample replication (Supplemental Figure S1). These samples were cross-dated with the QUB samples using ring-width or stable oxygen isotope dendrochronology (Supplemental Table S2). The chronology’s coverage is being constantly extended as further samples from the QUB English chronology, that had precise dates previously assigned to them, are processed and added to the developing record. All reported dates are currently dependent on the dendrochronological dating of the QUB English archive. Once complete, the third millennium BCE isotope reference chronology will be made publicly available. This will include all cross-matching statistics between the isotope series used in its construction. Additionally, the series will be made publicly accessible via ISODATE (Davies et al., 2025) for dating purposes. For the stable oxygen isotope analysis, the α-cellulose was extracted only from the latewood component of each oak sample, homogenized and freeze-dried (Loader et al., 1997; Wieloch et al., 2011). It was then weighed into silver capsules and pyrolized to carbon monoxide gas at 1400°C. The δ^18^O were measured on an isotope ratio mass spectrometer at Swansea University, UK. The measurements are reported in permil (‰) relative to the VSMOW standard (Coplen, 1995), with a typical analytical precision (σ_n−1_) of ±0.3 ‰ (Loader et al., 2013).The 15 oak δ^18^O series were indexed using a 9-year rectangular filter, and once securely cross-dated, averaged to produce the dating reference record. Following the statistical framework proposed by Loader et al. (2019), the agreement between the δ^18^O series was assessed using Pearson’s correlation coefficients *r*, Student’s *t*-values, probability of error estimates 1/*p*, and Isolation Factor values IF defined as the ratio of probabilities between the most and second most probable matches. A fit is indicated for consideration if 1/*p* ⩾ 100 and IF ⩾ 10 (Loader et al., 2019).

## Results

**Figure 2.**
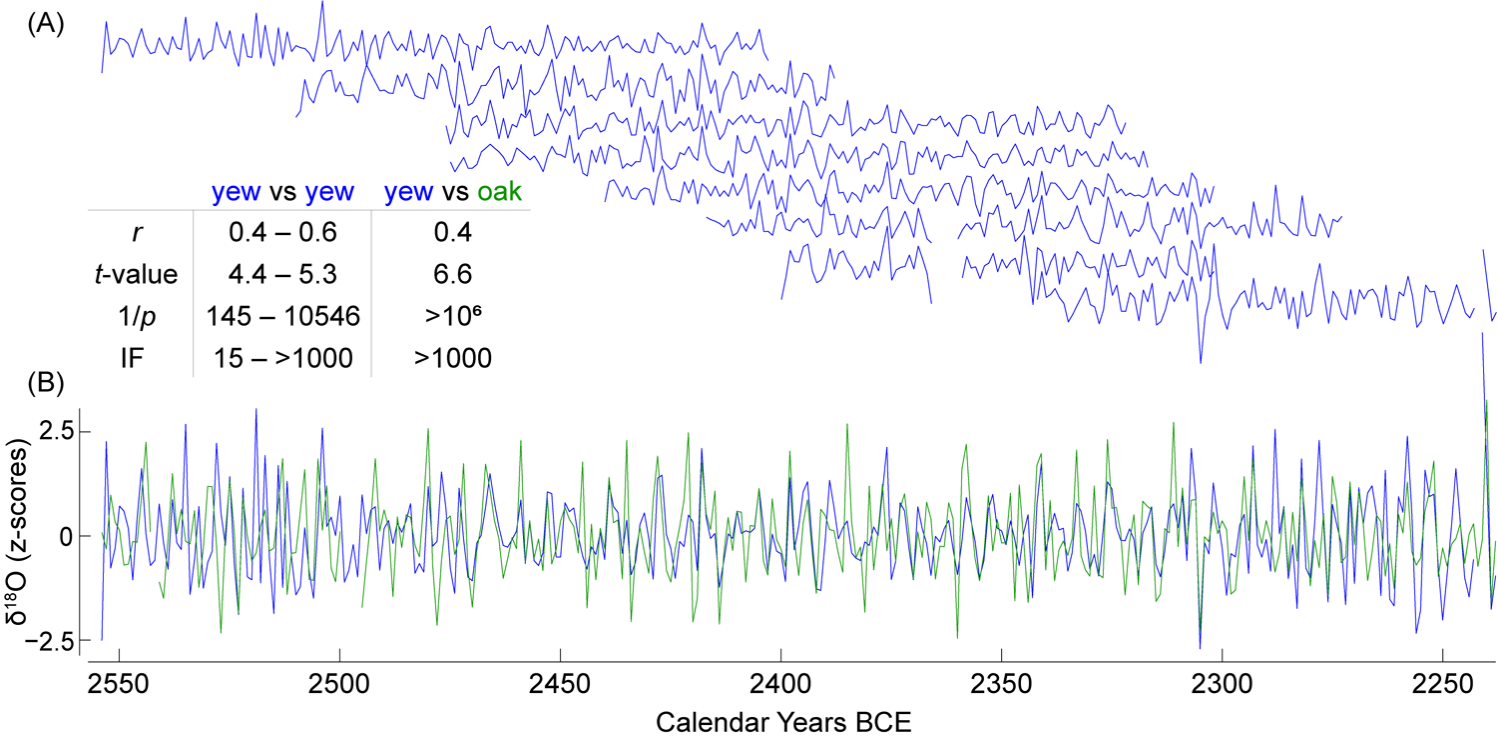
Cross-dating characteristics. (a) Eight individual yew δ^18^O series between which a statistically significant match is found, and that are used to develop a mean yew δ^18^O chronology, all detrended with a 9-year rectangular filter. Note that the gaps in data are due to a loss of samples at the lab. (b) Yew (blue) and reference oak (green) δ^18^O chronologies, both *z*-transformed. The inset table presents summary statistics of cross-dating the eight individual yew series against each other and their mean against the reference oak chronology: *r* is the Pearson’s correlation coefficient, *t* is the Student’s *t*-value, 1/*p* is the probability of error, IF is the Isolation Factor (for full statistics, see Supplemental Table S3 and Figure S2). A match is indicated for consideration if 1/*p* ⩾ 100 and IF ⩾ 10 (Loader et al., 2019). *Note*. Please refer to the online version of the article to view this figure in color.

Independent cross-dating of the 12 yew δ^18^O series against each other positions 8 of them in agreement with the original TRW-based cross-dating results. The 8 series correlate with each other with Pearson’s *r* values between 0.4 and 0.6, Student’s *t*-values between 4.4 and 5.3, probabilities of error 1/*p* values between 145 and 10,546 and Isolation Factor IF values between 15 and 1000 (Figure 2, Supplemental Table S3). The other four δ^18^O series do not pass the ISODATE statistical threshold for dating and currently remain undated, despite a high statistical agreement of these samples with the TRW master chronology (Supplemental Table S1). These results suggest that a lack of a strong limiting climate factor and a high percentage of locally absent rings in our yew wood samples (Bebchuk et al., 2024) complicate TRW cross-dating, and a multi-parameter/proxy approach should be used for dating verification. The eight δ^18^O series were crossmatched and merged in a stepwise manner into a single chronology spanning 311 years. Comparison between the yew and reference oak δ^18^O chronologies returns a statistically significant match with a correlation *r* of 0.37, Student’s *t*-value of 6.64, 1/*p* ⩾ 10^6^, and IF ⩾ 1000 (Figure 2, Supplemental Figure S2, Supplemental Table S3).

Intriguingly, our study presents the first example of oxygen isotopic agreement between oak and yew wood. We further emphasize that tree-ring stable isotopes can help overcome challenges associated with traditional TRW cross-dating, especially if a species has an irregular growth pattern, or tree growth is influenced by multiple climatic drivers and species exhibit different climate sensitivities. This approach can significantly advance archaeological and palaeoclimatic research, which are strongly limited by the availability of relict material.

**Figure 3.**
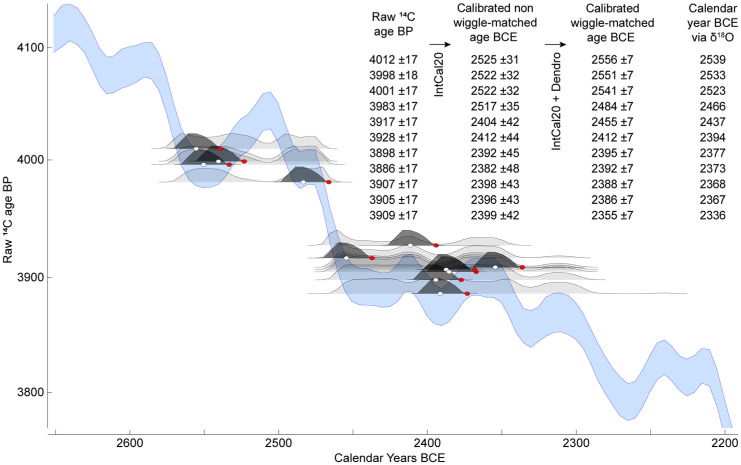
Radiocarbon and absolute dating. Raw results of radiocarbon dating are listed in the first column of the inset table (for full statistics, see Supplemental Table S4). The individual ^14^C dates are resolved against the most recent radiocarbon calibration curve for the Northern Hemisphere IntCal20 (in blue; Reimer et al., 2020) with their probability distributions shown in light grey and calibrated ages BCE given in the second column. To further reduce the temporal uncertainty, the dates are wiggle-matched using dendrochronological information (dark grey shadings and white dots for mean values, third column). Note that the ^14^C dates are integrated over 10–15 years, and this uncertainty is not taken into account in OxCal calibration (Bronk Ramsey, 2001, 2021). The calibrated dates are reported at the 95.4% probability. Absolute cross-dating of the yew δ^18^O chronology against the reference oak δ^18^O chronology assigns a calendar year BCE to each sample (red dots, last column). The difference between wiggle-matched and calendar dates (white and red dots, respectively) is 17 years. *Note*. Please refer to the online version of the article to view this figure in color.

Our absolute dating supports the results of the initial radiocarbon analysis (Figure 3, Supplemental Table S4). The calendar dates newly assigned to the 11 samples that were used for ^14^C measurements are within the 99.7% probability range given by wiggle-matching (Bronk Ramsey et al., 2001). Although the ^14^C values are slightly higher than the IntCal20 curve, especially across the plateau between circa 2450 and 2350 BCE, this discrepancy likely arises from both, samples integrating over 10–15 years and the calibration curve itself lacking a high temporal precision. Our study emphasizes the importance of increasing the total number of absolutely dated and annually resolved Holocene tree-ring chronologies, which may further help constrain radiocarbon calibration curves (Pearson et al., 2022).

**Figure 4.**
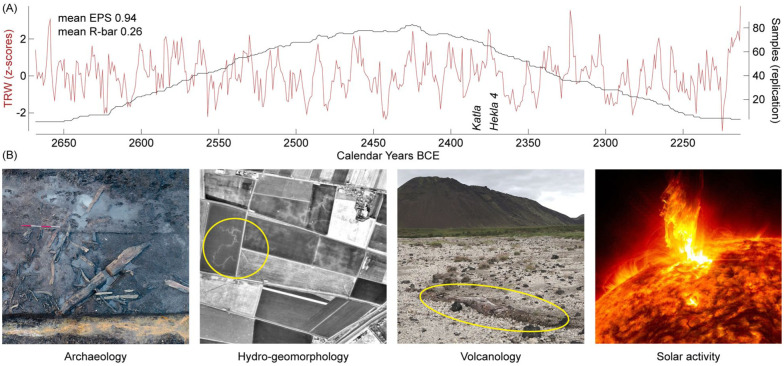
Chronology characteristics and applications. (a) Absolutely dated yew TRW chronology in red and its sample replication in black. EPS is the expressed population signal and *R*-bar is the inter-series correlation, both derived from 70-year windows lagged by 25 years from 2579–2309 BCE. Icelandic eruptions of Katla and Hekla 4 dated to 2388 ± 8 and 2375 ± 8 BCE, respectively, are labelled (Davies et al., 2024). (b) Potential applications of our new mid-Holocene record are: (i) dating archaeological finds of the Neolithic and Bronze Age periods (wood exposed during hand excavation of an area of Late Neolithic–Early Bronze Age lower peat at the archaeological site of Must Farm in the Fenland, UK; credits: Cambridge Archaeological Unit), (ii) reconstructing hydro-geomorphological and sea-level changes that may offer new insights into the yet debated 4.2 ka climate anomaly around 4200 years ago (paleo-streams encircled yellow, so-called roddons (Fowler, 1932), are visible next to the sampling site with subfossil yew wood; credits: Cambridge Air Photos, 1982; reproduced with permission of the Cambridge University Collection of Aerial Photography (c) Copyright reserved), (iii) dating volcanic eruptions (a tree trunk encircled yellow was buried by an Icelandic eruption some four thousand years ago; credits: UB), and (iv) reconstructing solar activity via annually resolved ^14^C measurements (a sudden explosion on the Sun generating a solar storm; credits: NASA’s Solar Dynamics Observatory).

The statistically significant inter-species isotopic agreement now places our yew TRW chronology precisely from 2668 to 2213 years BCE (Figure 4a). By establishing an absolute date, we secure a benchmark for further improvement of Fenland dendrochronology, add a new species into the limited list of high-resolution Holocene chronologies, and enable additional investigations of long tree-ring chronologies from Ireland (Baillie and Brown, 1988; Brown et al., 1986), Fennoscandia (Eronen et al., 2002; Grudd et al., 2002; Helama et al., 2008), and western Europe (Boettger et al., 2007; Eckstein et al., 2009; Leuschner et al., 2002).

## Discussion

Spanning the transition from the Late Neolithic to the Early Bronze Age, our absolutely dated yew chronology offers new opportunities for refining archaeological interpretation (Figure 4b). Systematic archaeological surveys in eastern England have unveiled vast and well-preserved evidence of early human activity (Godwin, 1997; Hall and Coles, 1994). Numerous houses, causewayed enclosures, trackways, and burial round borrows have been found (Medlycott 2011). Moreover, the period is characterized by the spread of metal-work and a remarkably wide range of pottery styles across the Fenland attributed to the Peterborough, Grooved Ware, and Beaker cultures (Bullmore, 2022; Glazebrook, 1997; Yates and Bradley, 2010). Despite a seemingly uniform landscape, regional diversity in archaeological findings has always been recognized, and developing a more precise spatiotemporal framework has consistently been identified as a research priority (Brown and Glazebrook, 2000; Cooper, 2024; Glazebrook, 1997; Medlycott, 2011. The calendar-dated yew TRW record now offers an opportunity for precise archaeological attribution.

The mid-Holocene hydroclimate reconstruction recently developed from Fenland yew wood (Bebchuk et al., 2025) is now anchored to the period 2645−2228 BCE. Further to shedding new light onto the hydro-geomorphological and biogeographic consequences of sea-level changes in the North Sea (Figure 4b) and their possible impacts on human migration and societal transformation, the record secures a timescale for high-resolution palaeoclimatic research on the still debated climate anomaly around 4200 years ago, the so-called ‘4.2 ka event’ (Helama, 2024; Weiss et al., 1993). Associated with an extreme drought in central Asia, its effect and extent across the North Atlantic remain unclear (Bradley and Bakke, 2019; McKay et al., 2024). Several lines of evidence point to increased storminess (Goslin et al., 2019; Sorrel et al., 2012) and a sea-level rise in the North Sea (Bebchuk et al., 2024, 2025; Shennan et al., 2018; Waller, 1994), which could have shifted (or be triggered by the shift of) the Inter-Tropical Convergence Zone southwards and contributed to drying across parts of Asia (Yan and Liu, 2019) and the European Alps (Arosio et al., 2025). We expect our yew archive to serve as a reference in advancing the quest for the causes and mechanisms of the 4.2 ka event.

Furthermore, our new absolutely dated yew chronology may help establish calendar dates for Icelandic volcanic eruptions, such as those of Katla and Hekla 4 that are currently estimated at 2388 ± 8 and 2375 ± 8 BCE, respectively (Figure 4b; Davies et al., 2024). This far distant dating might be possible through wood anatomical investigations of ‘blue rings’ that are known to occur in coniferous species and to be indicative of abrupt summer cooling often caused by large, sulphur-rich volcanic eruptions (Büntgen et al., 2022; Piermattei et al., 2015). We also expect many absolutely dated and annually resolved radiocarbon dates from our still growing yew archive to contribute to the refinement of the next IntCal curve (Reimer et al., 2020), and thus help reconstruct changes in solar activity during the mid-Holocene (Figure 4b). In the light of these opportunities, we hope that our study will stimulate measurements of tree-ring stable isotopes in relict wood across different species and regions and showcase their potential for advancing archaeological and paleoenvironmental research worldwide.

## Supplemental Material

sj-docx-1-hol-10.1177_09596836251407634 – Supplemental material for An absolutely dated mid-Holocene English yew chronology offers new opportunities for archaeological and palaeoenvironmental research Supplemental material, sj-docx-1-hol-10.1177_09596836251407634 for An absolutely dated mid-Holocene English yew chronology offers new opportunities for archaeological and palaeoenvironmental research by Tatiana Bebchuk, Darren Davies, Neil J Loader, Otmar Urban, Tito Arosio, Lukas Wacker, Natálie Pernicová, Josef Čáslavský, Miroslav Trnka, Alexander V Kirdyanov, David Brown, Jan Esper, Clive Oppenheimer and Ulf Büntgen in The Holocene

## References

[bibr1-09596836251407634] Arosio T Leuenberger M Nicolussi K , et al. (2025) Tree-ring stable isotopes from the European Alps reveal long-term summer drying over the Holocene. Science Advances 11: eadr4161. 10.1126/sciadv.adr4161 PMC11970467 40184451

[bibr2-09596836251407634] Astbury AK (1958) The Black Fens. Cambridge: Golden Head Press.

[bibr3-09596836251407634] Baillie MGL Brown DM (1988) An overview of oak chronologies. In: Science and Arhaeology. Presented at the Science and Arhaeology, B.A.R. British Series 196, Glasgow, 1987. Glasgow.

[bibr4-09596836251407634] Bannister B Robinson WJ (1975) Tree-ring dating in archaeology. World Archaeology 7: 210–225.

[bibr5-09596836251407634] Batchelor CR Branch NP Carew T , et al. (2020) Middle-Holocene environmental change and archaeology in coastal wetlands: Further implications for our understanding of the history of Taxus woodland. Holocene 30: 300–314.

[bibr6-09596836251407634] Bebchuk T Krusic PJ Pike JH , et al. (2024) Sudden disappearance of yew (Taxus baccata) woodlands from eastern England coincides with a possible climate event around 4.2 ka ago. Quaternary Science Reviews 323: 108414.

[bibr7-09596836251407634] Bebchuk T Büntgen U (2025) Securing the past for the future – why climate proxy archives should be protected. Boreas 70039.

[bibr8-09596836251407634] Bebchuk T Urban O Arosio T , et al. (2025) Tree-ring stable isotopes reveal a hydroclimate shift in Eastern England around 4.2 ka ago. Geophysical Research Letters 52: e2024GL114313.

[bibr9-09596836251407634] Billamboz A (2002) NOAA/WDS Paleoclimatology - Billamboz - Upper Suevia Bog 4 Archaeological - QUSP - ITRDB GERM030. Available at: 10.25921/574E-XY23

[bibr10-09596836251407634] Billamboz A (2003) Tree Rings and Wetland Occupation in Southwest Germany Between 2000 and 500 BC: Dendroarchaeology Beyond Dating in Tribute to F. H. Schweingruber.

[bibr11-09596836251407634] Boettger T Haupt M Knöller K , et al. (2007) Wood cellulose preparation methods and mass spectrometric analyses of δ13C, δ18O, and nonexchangeable δ2H values in cellulose, sugar, and starch: An interlaboratory comparison. Analytical Chemistry 79: 4603–4612. 17503767 10.1021/ac0700023

[bibr12-09596836251407634] Boswijk IG (1998) A dendrochronological study of oak and pine from the raised mires of the Humberhead Levels, Eastern England. Ph.D. Thesis. Sheffield: The University of Sheffield.

[bibr13-09596836251407634] Bradley RS Bakke J (2019) Is there evidence for a 4.2 ka bp event in the Northern North Atlantic region? Climate of the Past 15: 1665–1676.

[bibr14-09596836251407634] Bronk Ramsey C (1995) Radiocarbon calibration and analysis of stratigraphy: The OxCal program. Radiocarbon 37: 425–430.

[bibr15-09596836251407634] Bronk Ramsey C (2001) Development of the radiocarbon calibration program. Radiocarbon 43: 355–363.

[bibr16-09596836251407634] Bronk Ramsey C (2021) OxCal v4. 4.4. Available at: https://c14.arch.ox.ac.uk/oxcal/OxCal.html.

[bibr17-09596836251407634] Bronk Ramsey C van der Plicht J Weninger B (2001) ‘Wiggle matching’ radiocarbon dates. Radiocarbon 43: 381–389.

[bibr18-09596836251407634] Brown DM Munro MAR Baillie MGL , et al. (1986) Dendrochronology—The absolute Irish standard. Radiocarbon 28: 279–283.

[bibr19-09596836251407634] Brown N Glazebrook J (eds) (2000) Research and Archaeology: A Framework for the Eastern Counties 2. Research Agenda and Strategy. Norwich: East Anglian Archaeology.

[bibr20-09596836251407634] Bullmore H (2022) Houses of the living: Domestic architecture in England and Wales, 4000-1500 BC. Doctoral Thesis. UCL, University College, London.

[bibr21-09596836251407634] Büntgen U Crivellaro A Arseneault D , et al. (2022) Global wood anatomical perspective on the onset of the Late Antique Little Ice Age (LALIA) in the mid-6th century CE. Science Bulletin 67: 2336–2344. 36546223 10.1016/j.scib.2022.10.019

[bibr22-09596836251407634] Büntgen U Esper J (2025) The need for high-resolution paleoclimate research. Dialogues Clim. Change 2: 18–25.

[bibr23-09596836251407634] Büntgen U Myglan VS Ljungqvist FC , et al. (2016) Cooling and societal change during the late antique little ice age from 536 to around 660 AD. Nature Geoscience 9: 231–236.

[bibr24-09596836251407634] Büntgen U Oppenheimer C (2020) The importance of “year zero” in interdisciplinary studies of climate and history. Proceedings of the National Academy of Sciences 117: 32845–32847. 10.1073/pnas.2018103117 PMC7776855 33293418

[bibr25-09596836251407634] Christopoulou A Özarslan Y Elzanowska A , et al. (2024) Dendroarchaeology in Greece – From humble beginnings to promising future. Dendrochronologia 85: 126196.

[bibr26-09596836251407634] Cook ER Kairiukstis LA (2013) Methods of Dendrochronology: Applications in the Environmental Sciences. Dordreacht: Springer Science & Business Media.

[bibr27-09596836251407634] Coplen TB (1995) Letter to the editor: New IUPAC guidelines for the reporting of stable hydrogen, carbon, and oxygen isotope-ratio data. Journal of Research of the National Institute of Standards and Technology 100: 285. 29151742 10.6028/jres.100.021 PMC4887246

[bibr28-09596836251407634] Darby HC (1940) The Draining of the Fens. Cambridge: Cambridge University Press.

[bibr29-09596836251407634] Darby HC (1983) The Changing Fenland. Cambridge: Cambridge University Press.

[bibr30-09596836251407634] Davies D Loader NJ McCarroll D , et al. (2025) ISODATE – Software for stable isotope dendrochronology. Dendrochronologia 93: 126385.

[bibr31-09596836251407634] Davies SM Albert PG Bourne AJ , et al. (2024) Exploiting the Greenland volcanic ash repository to date caldera-forming eruptions and widespread isochrons during the Holocene. Quaternary Science Reviews 334: 108707.

[bibr32-09596836251407634] Douglass AE (1941) Crossdating in dendrochronology. Journal of Forestry 39: 825–831.

[bibr33-09596836251407634] D’Arrigo R Frank D Jacoby G , et al. (2001) Spatial response to major volcanic events in or about AD 536, 934 and 1258: Frost rings and other dendrochronological evidence from Mongolia and Northern Siberia: Comment on R. B. Stothers, ‘Volcanic Dry Fogs, Climate Cooling, and Plague Pandemics in Europe and the Middle East’ (Climatic Change, 42, 1999). Climatic Change 49: 239–246.

[bibr34-09596836251407634] Cooper A (2024) Early and Middle Bronze Age Resource Assessment (2024) In: Research and archaeology: East of England research framework. Available at: https://researchframeworks.org/eoe/resource-assessments/early-and-middle-bronze-age/#section-13

[bibr35-09596836251407634] Eckstein J Leuschner HH Bauerochse A , et al. (2009) Subfossil bog-pine horizons document climate and ecosystem changes during the mid-Holocene. Dendrochronologia 27: 129–146.

[bibr36-09596836251407634] Ennion EAR (1951) Cambridgeshire, Huntingdonshire and the Isle of Ely. London: Hale.

[bibr37-09596836251407634] Eronen M Zetterberg P Briffa KR , et al. (2002) The supra-long Scots pine tree-ring record for Finnish lapland: Part 1, chronology construction and initial inferences. Holocene 12: 673–680.

[bibr38-09596836251407634] Esper J Krusic PJ Ljungqvist FC , et al. (2016) Ranking of tree-ring based temperature reconstructions of the past millennium. Quaternary Science Reviews 145: 134–151.

[bibr39-09596836251407634] Esper J Schneider L Krusic PJ , et al. (2013) European summer temperature response to annually dated volcanic eruptions over the past nine centuries. Bulletin of Volcanology 75: 736.

[bibr40-09596836251407634] Fowler G (1932) Old river-beds in the Fenlands. Geographical Journal 79: 210–212.

[bibr41-09596836251407634] Fritts HC (1976) Tree Rings and Climate. Tucson, Arizona, USA: Academic Press, Laboratory of Tree-Ring Researh, University of Arizona.

[bibr42-09596836251407634] Glazebrook J (ed.) (1997) Research and archaeology: A framework for the Eastern Counties 1. Resource assessment. Norwich: East Anglian Archaeology.

[bibr43-09596836251407634] Godwin H (1978) Fenland: its ancient past and uncertain future. Cambridge: Cambridge University Press.

[bibr44-09596836251407634] Godwin H (1997) The contribution of radiocarbon dating to archaeology in Britain. Philosophical Transactions of the Royal Society A (Mathematics, Physical and Engineering Sciences) 269: 57–75.

[bibr45-09596836251407634] Goslin J Gałka M Sander L , et al. (2019) Decadal variability of North-Eastern Atlantic storminess at the mid-Holocene: New inferences from a record of wind-blown sand, Western Denmark. Global and Planetary Change 180: 16–32.

[bibr46-09596836251407634] Grudd H Briffa KR Karlén W , et al. (2002) A 7400-year tree-ring chronology in Northern Swedish Lapland: Natural climatic variability expressed on annual to millennial timescales. Holocene 12: 657–665.

[bibr47-09596836251407634] Hafner A Reich J Ballmer A , et al. (2021) First absolute chronologies of neolithic and bronze age settlements at Lake Ohrid based on dendrochronology and radiocarbon dating. Journal of Archaeological Science Reports 38: 103107.

[bibr48-09596836251407634] Hall D Coles J (eds) (1994) Fenland Survey: An Essay in Landscape and Persistence, dgo-digital original edn. Liverpool: Liverpool University Press.

[bibr49-09596836251407634] Haneca K Debonne V Davies D , et al. (2025) Oxygen isotope dendrochronology allows dating of historical timbers across a wide geographical region. Dendrochronologia 89: 126283.

[bibr50-09596836251407634] Hartl-Meier C Zang C Buntgen U , et al. (2015) Uniform climate sensitivity in tree-ring stable isotopes across species and sites in a mid-latitude temperate forest. Tree Physiology 35: 4–15. 25466725 10.1093/treephys/tpu096

[bibr51-09596836251407634] Helama S (2024) The 4.2 ka event: A review of palaeoclimate literature and directions for future research. Holocene 34: 1408–1415.

[bibr52-09596836251407634] Helama S Mielikäinen K Timonen M , et al. (2008) Finnish supra-long tree-ring chronology extended to 5634 BC. Norsk Geografisk Tidsskrift - Norwegian Journal of Geography 62: 271–277.

[bibr53-09596836251407634] Holmes RL (1983) Computer-assisted quality control in tree-ring dating and measurement. TREE-RING Bull 43: 69–78.

[bibr54-09596836251407634] Kuniholm PI Newton M Sherbiny H , et al. (2014) Dendrochronological dating in Egypt: Work accomplished and future prospects. Radiocarbon 56: S93–S102.

[bibr55-09596836251407634] Leuschner HH Sass-Klaassen U Jansma E , et al. (2002) Subfossil European bog oaks: Population dynamics and long-term growth depressions as indicators of changes in the holocene hydro-regime and climate. Holocene 12: 695–706.

[bibr56-09596836251407634] Ljungqvist FC Piermattei A Seim A , et al. (2020) Ranking of tree-ring based hydroclimate reconstructions of the past millennium. Quaternary Science Reviews 230: 106074.

[bibr57-09596836251407634] Loader NJ Mccarroll D Miles D , et al. (2019) Tree ring dating using oxygen isotopes: A master chronology for Central England. Journal of Quaternary Science 34: 475–490.

[bibr58-09596836251407634] Loader NJ McCarroll D Miles D , et al. (2021) Dating of non-oak species in the United Kingdom historical buildings archive using stable oxygen isotopes. Dendrochronologia 69: 125862.

[bibr59-09596836251407634] Loader NJ Robertson I Barker AC , et al. (1997) An improved technique for the batch processing of small wholewood samples to α-cellulose. Chemical Geology 136: 313–317.

[bibr60-09596836251407634] Loader NJ Santillo PM Woodman-Ralph JP , et al. (2008) Multiple stable isotopes from oak trees in Southwestern Scotland and the potential for stable isotope dendroclimatology in maritime climatic regions. Chemical Geology 252: 62–71.

[bibr61-09596836251407634] Loader NJ Young GHF McCarroll D , et al. (2013) Quantifying uncertainty in isotope dendroclimatology. Holocene 23: 1221–1226.

[bibr62-09596836251407634] McCarroll D Loader NJ (2004) Stable isotopes in tree rings. Isotopes in Quaternary Paleoenvironmental reconstruction 23: 771–801.

[bibr63-09596836251407634] McCarroll D Loader NJ Miles D , et al. (2019) Oxygen isotope dendrochronology of Llwyn Celyn; one of the oldest houses in Wales. Dendrochronologia 58: 125653.

[bibr64-09596836251407634] McKay NP Kaufman DS Arcusa SH , et al. (2024) The 4.2 ka event is not remarkable in the context of holocene climate variability. Nature Communications 15: 6555. 10.1038/s41467-024-50886-w PMC11297131 39095415

[bibr65-09596836251407634] Medlycott M (ed.)(2011) Research and Archaeology Revisited: A Revised Framework for the East of England. Norwich: East Anglian Archaeology.

[bibr66-09596836251407634] Miller SH Skertchly SBJ (1878) The Fenland Past and Present. London: Leach and Son.

[bibr67-09596836251407634] Nakatsuka T Sano M Li Z , et al. (2020) A 2600-year summer climate reconstruction in Central Japan by integrating tree-ring stable oxygen and hydrogen isotopes. Climate of the Past 16: 2153–2172.

[bibr68-09596836251407634] Nayling N Loader NJ Bale RJ , et al. (2024a) Inter-genus oxygen isotope dendrochronology of the Newport medieval ship keel. International Journal of Nautical Archaeology 53: 535–540.

[bibr69-09596836251407634] Nayling N Loader NJ Bale RJ , et al. (2024b) Oxygen isotope dendrochronology of the Newport medieval ship. International Journal of Nautical Archaeology 53: 245–253.

[bibr70-09596836251407634] Němec M Wacker L Hajdas I , et al. (2010) Alternative methods for cellulose preparation for AMS Measurement. Radiocarbon 52: 1358–1370.

[bibr71-09596836251407634] Oppenheimer C Orchard A Stoffel M , et al. (2018) The Eldgjá eruption: Timing, long-range impacts and influence on the Christianisation of Iceland. Climatic Change 147: 369–381. 31258223 10.1007/s10584-018-2171-9 PMC6560931

[bibr72-09596836251407634] Pearson C Salzer M Wacker L , et al. (2020) Securing timelines in the ancient Mediterranean using multiproxy annual tree-ring data. Proceedings of the National Academy of Sciences 117: 8410–8415. 10.1073/pnas.1917445117 PMC7165418 32229554

[bibr73-09596836251407634] Pearson CL Leavitt SW Kromer B , et al. (2022) Dendrochronology and radiocarbon dating. Radiocarbon 64: 569–588.

[bibr74-09596836251407634] Piermattei A Crivellaro A Carrer M , et al. (2015) The “blue ring”: Anatomy and formation hypothesis of a new tree-ring anomaly in conifers. Trees 29: 613–620.

[bibr75-09596836251407634] Pilcher JR Baillie MGL Schmidt B , et al. (1984) A 7,272-year tree-ring chronology for Western Europe. Nature 312: 150–152.

[bibr76-09596836251407634] Pryme ADL (1701) III. Part of a letter from the Reverend Mr Abraham de la Pryme to the publisher, concerning trees found under ground in Hatfield Chace *Philos*. Trans 1683-1775 2222: 980–992.

[bibr77-09596836251407634] Pryor F (2019) The Fens: Discovering England’s Ancient Depths. London: Bloomsbury Publishing.

[bibr78-09596836251407634] Reimer PJ Austin WEN Bard E , et al. (2020) The IntCal20 Northern Hemisphere radiocarbon age calibration curve (0–55 cal kBP). Radiocarbon 62: 725–757.

[bibr79-09596836251407634] Reinig F Sookdeo A Esper J , et al. (2020) Illuminating intcal during the younger dryas. Radiocarbon 62: 883–889.

[bibr80-09596836251407634] Rinn F (1996) TSAP - Time Series Analyses Presentation. Reference Manual (Version 3.0). Heidelberg: RinnTech Heidelb.

[bibr81-09596836251407634] Römer P Reinig F Konter O , et al. (2023) Multi-proxy crossdating extends the longest high-elevation tree-ring chronology from the Mediterranean. Dendrochronologia 79: 126085.

[bibr82-09596836251407634] Sano M Li Z Murakami Y , et al. (2022) Tree ring oxygen isotope dating of wood recovered from a canal in the ancient capital of Japan. Journal of Archaeological Science Reports 45: 103626.

[bibr83-09596836251407634] Shennan I Bradley SL Edwards R (2018) Relative sea-level changes and crustal movements in Britain and Ireland since the Last Glacial Maximum. Quaternary Science Reviews 188: 143–159.

[bibr84-09596836251407634] Shi S Shi J Nakatsuka T , et al. (2025) Tree-ring oxygen isotope cross-dating between southeastern China and central Japan. Dendrochronologia 91: 126319.

[bibr85-09596836251407634] Siegwolf RTW Brooks JR Roden J , et al. (2022) Stable Isotopes in Tree Rings: Inferring Physiological, Climatic and Environmental Responses, Tree Physiology. Cham: Springer International Publishing.

[bibr86-09596836251407634] Skertchly SBJ (1877) The geology of the Fenland. Order of the Lords Commissioners of Her Majesty’s Treasury. London.

[bibr87-09596836251407634] Sookdeo A Kromer B Büntgen U , et al. (2020) Quality dating: A well-defined protocol implemented at ETH for high-precision 14C-dates tested on late glacial wood. Radiocarbon 62: 891–899.

[bibr88-09596836251407634] Sorrel P Debret M Billeaud I , et al. (2012) Persistent non-solar forcing of Holocene storm dynamics in coastal sedimentary archives. Nature Geoscience 5: 892–896.

[bibr89-09596836251407634] Tegel W Muigg B Skiadaresis G , et al. (2022) Dendroarchaeology in Europe. Frontiers in Ecology and Evolution 10: 823622.

[bibr90-09596836251407634] Wacker L Bonani G Friedrich M , et al. (2010) MICADAS: Routine and high-precision radiocarbon dating. Radiocarbon 52: 252–262.

[bibr91-09596836251407634] Waller M (1994) The Fenland Project, Number 9: Flandrian Environmental Change in Fenland, East Anglian Archaeology. Cambridge: Cambridgeshire Archaeological Committee.

[bibr92-09596836251407634] Weiss H Courty M-A Wetterstrom W , et al. (1993) The Genesis and collapse of third millennium North Mesopotamian civilization. Science 261: 995–1004. 17739617 10.1126/science.261.5124.995

[bibr93-09596836251407634] Wieloch T Helle G Heinrich I , et al. (2011) A novel device for batch-wise isolation of α-cellulose from small-amount wholewood samples. Dendrochronologia 29: 115–117.

[bibr94-09596836251407634] Wigley TML Jones PD Briffa KR (1987) Cross-dating methods in dendrochronology. Journal of Archaeological Science 14: 51–64.

[bibr95-09596836251407634] Yan M Liu J (2019) Physical processes of cooling and mega-drought during the 4.2 ka BP event: Results from TraCE-21ka simulations. Climate of the Past 15: 265–277.

[bibr96-09596836251407634] Yates D Bradley R (2010) Still water, hidden depths: The deposition of Bronze Age metalwork in the English Fenland. Antiquity 84: 405–415.

