# Supplementary material for: An absolutely dated mid-Holocene English yew chronology offers new opportunities for archaeological and palaeoenvironmental research
Source: Holocene. 2026 Feb 3;36(4):382–9. doi: 10.1177/09596836251407634 (PMC12974950; doi:10.1177/09596836251407634)
Supplement: sj-docx-1-hol-10.1177_09596836251407634 – Supplemental material for An absolutely dated mid-Holocene English yew chronology offers new opportunities for archaeological and palaeoenvironmental research [file sj-docx-1-hol-10.1177_09596836251407634.docx]

Supplementary material for

**An absolutely dated English yew chronology for the mid-Holocene offers new opportunities for archaeological and paleoenvironmental research**

**Table S1.** Cross-dating statistics for 100 yew tree-ring width measurement series. The series highlighted are the samples for which δ^18^O were analysed. The eight (four) series in green (yellow) passed (did not pass) statistical thresholds set by the ISODATE software for consideration.

Seq Series Time_span 620 640 660 680 700 720 740 760 780 800 820 840 860 880 900 920 940 960 980 1000

694 714 734 754 774 794 814 834 854 874 894 914 934 954 974 994 1014 1034 1054 1074

--- -------- --------- ---- ---- ---- ---- ---- ---- ---- ---- ---- ---- ---- ---- ---- ---- ---- ---- ---- ---- ---- ----

1 F1TB375M 611 863 .33 .42 .59 .62 .64 .65 .58 .56 .40 .39

2 F1TB351M 630 799 .34 .44 .45 .36 .44 .45 .44

3 F2TB011m 635 861 .39 .38 .30 .35 .45 .49 .58 .58 .58 .38

4 F1TB009m 636 764 .37 .37 .52 .47 .42

5 F1TB369M 639 862 .35 .46 .51 .48 .48 .38 .46 .42 .55 .51

6 F2TB021m 640 772 .23A .56 .61 .52

7 F2TB028m 646 799 .51 .45 .44 .40 .26A .28

8 F1TB074M 659 828 .43 .44 .40 .47 .51 .53 .61

9 F1TB096m 659 809 .48 .48 .58 .60 .53 .54

10 F1TB338N 660 899 .50 .52 .54 .48 .43 .38 .39 .41 .32 .31

11 F1TB478M 660 933 .31 .33 .48 .47 .56 .55 .49 .37 .26A .35 .34

12 F1TB075m 661 900 .40 .30B .29B .34 .26A .47 .45 .43 .43 .41

13 F1TB448M 662 864 .62 .70 .74 .79 .62 .54 .39 .37

14 F1TB028m 663 732 .52

15 F1TB479M 668 924 .46 .53 .52 .50 .50 .56 .63 .57 .55 .44 .40

16 F1TB463M 669 889 .52 .64 .70 .43 .41 .35 .36 .40 .31

17 F1TB368M 670 879 .61 .66 .60 .63 .59 .56 .52 .43 .41

18 F1TB404N 673 855 .46 .53 .54 .55 .47 .49 .34 .35

19 F1TB323M 674 886 .60 .59 .70 .73 .70 .73 .74 .64 .55

20 F1TB089m 675 770 .46 .49 .51

21 F1TB091B 676 814 .60 .63 .62 .63 .54

22 F1TB063m 677 837 .37 .37 .38 .50 .35 .36 .30

23 F1TB358N 680 919 .59 .64 .58 .54 .54 .62 .58 .51 .38 .33

24 F1TB326M 682 956 .52 .60 .67 .73 .77 .66 .53 .61 .55 .56 .47 .46

25 F1TB081m 686 796 .57 .54 .55 .55

26 F1TB056M 687 940 .44 .59 .58 .52 .59 .63 .58 .58 .46 .37 .37

27 F1TB366M 690 857 .43 .47 .52 .49 .52 .48 .46

28 F1TB344M 695 859 .58 .62 .52 .36 .16B .33 .34

29 F1TB355M 695 975 .69 .74 .74 .71 .73 .78 .74 .75 .72 .65 .55 .47 .46

30 F1TB012m 696 877 .47 .46 .49 .51 .56 .62 .58 .54

31 F1TB321A 699 896 .47 .46 .46 .34 .39 .45 .50 .54 .52

32 F1TB328M 700 907 .42 .62 .75 .73 .74 .64 .62 .52

33 F1TB437M 703 859 .63 .62 .64 .53 .30B .31B

34 F1TB076m 704 794 .57 .55

35 F1TB002m 705 915 .69 .76 .62 .64 .59 .43 .37 .28 .27A

36 F1TB412N 706 899 .45 .53 .69 .71 .69 .63 .52 .45

37 F1TB023m 711 881 .65 .74 .76 .64 .67 .44 .45

38 F1TB100m 712 854 .40 .45 .59 .69 .60

39 F1TB360M 712 923 .60 .60 .68 .79 .80 .73 .72 .53 .49

40 F2TB001m 713 859 .43 .51 .53 .54 .42 .38

41 F1TB373M 716 887 .44 .50 .62 .69 .63 .58 .63

42 F1TB457N 716 950 .42 .45 .60 .45 .41 .24A .31 .35 .41 .48

43 F1TB445M 723 949 .52 .52 .50 .48 .51 .60 .58 .60 .63

44 F1TB325N 727 1059 .51 .61 .62 .57 .56 .60 .73 .75 .77 .67 .67 .61 .40 .26B .20B

45 F1TB057m 729 893 .50 .42 .42 .42 .38 .48

46 F2TB002m 731 913 .56 .64 .67 .60 .67 .58 .55

47 F2TB005m 733 904 .62 .65 .71 .75 .64 .66 .64

48 F1TB043C 735 907 .46 .48 .51 .48 .37 .51 .55

49 F1TB203M 735 947 .35 .50 .62 .51 .50 .46 .42 .40 .40

50 F1TB353M 736 906 .64 .72 .77 .66 .64 .45 .34

51 F1TB354M 738 942 .56 .56 .57 .61 .62 .66 .61 .56 .53

52 F1TB451M 739 1015 .50 .50 .69 .73 .73 .68 .59 .58 .61 .60 .56 .52 .50

53 F1TB004m 740 968 .57 .54 .48 .42 .41 .50 .52 .37 .43

54 F1TB035M 740 945 .13B .47 .45 .60 .61 .62 .70 .68

55 F1TB306M 740 971 .62 .75 .77 .77 .76 .53 .56 .48 .33

56 F1TB339N 742 929 .64 .63 .56 .46 .32 .31 .39

57 F1TB335m 748 969 .57 .61 .55 .40 .42 .40 .42 .49 .40

58 F1TB372M 750 929 .47 .55 .60 .54 .53 .44 .39

59 F1TB007m 753 949 .64 .66 .56 .39 .31B .26B .34 .38

60 F1TB336M 755 989 .55 .57 .51 .47 .39 .34 .48 .46 .40 .32

61 F1TB310M 758 971 .62 .61 .54 .50 .53 .46 .46 .46 .36

62 F1TB077m 760 944 .47 .53 .47 .42 .34B .40 .35

63 F1TB017m 761 911 .55 .62 .62 .50 .32

64 F1TB003m 762 939 .55 .69 .64 .65 .68 .52 .50

65 F1TB038m 765 934 .48 .59 .60 .60 .63 .50

66 F1TB020M 766 907 .42 .65 .64 .62 .57

67 F1TB008M 768 1025 .59 .65 .70 .72 .73 .73 .64 .59 .54 .34 .35

68 F1TB026M 772 909 .55 .57 .55 .55 .38

69 F1TB349C 773 929 .45 .49 .40 .48 .46 .35

70 F1TB045m 776 994 .58 .62 .59 .42 .33 .27 .23A .28 .34B

71 F1TB426N 776 999 .61 .61 .56 .65 .66 .64 .64 .67 .55 .51

72 F1TB329M 777 989 .73 .76 .71 .63 .52 .53 .47 .43 .40

73 F1TB359M 789 989 .75 .68 .58 .53 .53 .50 .45 .38

74 F1TB332M 790 1066 .35 .27 .46 .48 .63 .73 .75 .63 .44 .22B .25A .28

75 F1TB315M 792 919 .37 .46 .63 .56 .59

76 F1TB060m 793 939 .63 .62 .59 .49 .56 .54

77 F1TB423B 793 1019 .40 .38 .44 .56 .55 .56 .44 .52 .45 .42

78 F1TB314m 794 1009 .36 .42 .60 .59 .63 .62 .64 .55 .40

79 F1TB095m 797 967 .43 .50 .57 .50 .50 .44 .39

80 F1TB308M 801 940 .40 .51 .49 .55 .50

81 F1TB334M 803 1003 .33 .45 .56 .51 .43 .38 .42 .36

82 F1TB066m 805 929 .67 .63 .59 .61

83 F2TB020M 805 1029 .53 .50 .47 .47 .50 .55 .54 .54 .52

84 F1TB313M 807 987 .49 .50 .42 .53 .53 .55 .50

85 F1TB367M 815 1023 .52 .58 .61 .63 .61 .58 .59 .53 .54

86 F1TB383M 817 1039 .62 .66 .59 .56 .50 .41 .39 .39 .34 .29B

87 F1TB496M 817 1011 .42 .42 .46 .57 .62 .66 .60 .55

88 F1TB048m 819 1011 .41 .42 .42 .52 .61 .70 .61 .53

89 F1TB361M 836 993 .29 .41 .54 .56 .45 .49

90 F1TB333N 845 989 .41 .48 .59 .52 .37

91 F1TB050m 847 1035 .47 .51 .55 .37 .40 .39 .34 .36

92 F2TB031m 847 976 .42 .58 .61 .54 .56

93 F1TB086m 850 1028 .61 .73 .73 .69 .73 .64 .55

94 F1TB381M 850 1007 .28 .43 .56 .54 .54 .45

95 F1TB085m 854 1020 .45 .44 .50 .54 .64 .64 .66

96 F1TB341M 856 1022 .32 .33 .49 .54 .58 .42 .40

97 F1TB311M 871 1067 .38 .47 .55 .66 .46 .28 .15B .31

98 F1TB054m 886 1035 .62 .60 .60 .55 .42 .43

99 BFTB002N 935 1020 .52 .57 .51

100 F1TB319A 990 1067 .45 .52

Av segment correlation .36 .42 .49 .51 .53 .54 .54 .57 .55 .51 .52 .48 .50 .53 .51 .52 .49 .43 .31 .33

**Table S2.** Stable oxygen isotope cross-dating results of the 4 additional oak samples against a reference chronology built using only precisely dated timbers from the English bog oak chronology constructed at Queen’s University Belfast. Match is the astronomical year of the most probable match, *n* is the number of data pairs (i.e., the overlap length), *r* is the Pearson’s correlation coefficient, *df* is the corrected degrees of freedom, *t* is the Student’s *t*-value, 1/*p* is the probability of error, IF is the Isolation Factor, and Pass indicates whether a match passes the statistical thresholds (1/*p* ≥ 100 and IF ≥ 10) for consideration. A minimum overlap of 20 pairs of data was set. Cross-dating was performed using the ISODATE software (Davies et al., 2025).

| Sample | Match | *n* | *r* | *df* | *t* | 1/*p* | IF | Pass |
| --- | --- | --- | --- | --- | --- | --- | --- | --- |
| FEN_01 | -2640 | 86 | 0.70 | 74 | 8.53 | ≥ 10^6^ | ≥ 1000 | TRUE |
| FEN_04 | -2819 | 90 | 0.69 | 77 | 8.37 | ≥ 10^6^ | ≥ 1000 | TRUE |
| JOMF_2 | -2638 | 110 | 0.63 | 95 | 7.88 | ≥ 10^6^ | ≥ 1000 | TRUE |
| LNWD_01 | -2837 | 49 | 0.73 | 41 | 6.91 | 83844 | ≥ 1000 | TRUE |

**Table S3.** Statistical parameters of the eight individual yew δ^18^O series cross-dated against each other and their mean (Yew_8) cross-dated against the reference oak chronology: *n* is the number of data pairs (i.e., the overlap length), *r* is the Pearson’s correlation coefficient, *df* is the corrected degree of freedom, *t* is the Student’s *t*-value, 1/*p* is the probability of error, IF is the Isolation Factor, and Pass indicates whether a match passes the statistical thresholds (1/*p* ≥ 100 and IF ≥ 10) to be indicated for consideration.

| Sample | Reference | *n* | *r* | *df* | *t* | 1/*p* | IF | Pass |
| --- | --- | --- | --- | --- | --- | --- | --- | --- |
| 1 | 2 | 119 | 0.46 | 103 | 5.27 | 10546 | ≥1000 | TRUE |
| 3 | 1_2 | 93 | 0.46 | 80 | 4.68 | 748 | 425 | TRUE |
| 4 | 1_2_3 | 111 | 0.45 | 96 | 5 | 2732 | ≥1000 | TRUE |
| 5 | 1_2_3_4 | 70 | 0.49 | 60 | 4.36 | 145 | 15 | TRUE |
| 6 | 1_2_3_4_5 | 159 | 0.4 | 137 | 5.12 | 5565 | 18 | TRUE |
| 7 | 1_2_3_4_5_6 | 74 | 0.56 | 60 | 5.3 | 3218 | 60 | TRUE |
| 8 | 1_2_3_4_5_6_7 | 123 | 0.42 | 106 | 4.8 | 958 | ≥1000 | TRUE |
| Yew_8 | Oak | 311 | 0.37 | 273 | 6.64 | ≥10^6^ | ≥1000 | TRUE |

**Table S4.** Raw radiocarbon (^14^C) dates reported in age Before Present (BP) and calendar dates derived via δ^18^O dating reported in years Before Common Era (BCE; datum includes the year zero).

| Lab code | δ^13^C (‰) | Raw ^14^C age BP | ^14^C age σ | Year BCE |
| --- | --- | --- | --- | --- |
| ETH 111184.1.1 | -23.5 | 4,012 | 17 | 2539 |
| 54891 | -22.2 | 3,998 | 18 | 2533 |
| ETH 111180.1.1 | -21.3 | 4,001 | 17 | 2523 |
| ETH 111179.1.1 | -25.2 | 3,983 | 17 | 2466 |
| ETH 111182.1.1 | -21.7 | 3,917 | 17 | 2437 |
| ETH 111172.1.1 | -21.5 | 3,928 | 17 | 2394 |
| ETH 111174.1.1 | -22.8 | 3,898 | 17 | 2377 |
| ETH 111183.1.1 | -20.9 | 3,886 | 17 | 2373 |
| ETH 111177.1.1 | -21.4 | 3,907 | 17 | 2368 |
| ETH 111173.1.1 | -24.0 | 3,905 | 17 | 2367 |
| ETH 111178.1.1 | -21.8 | 3,909 | 17 | 2336 |


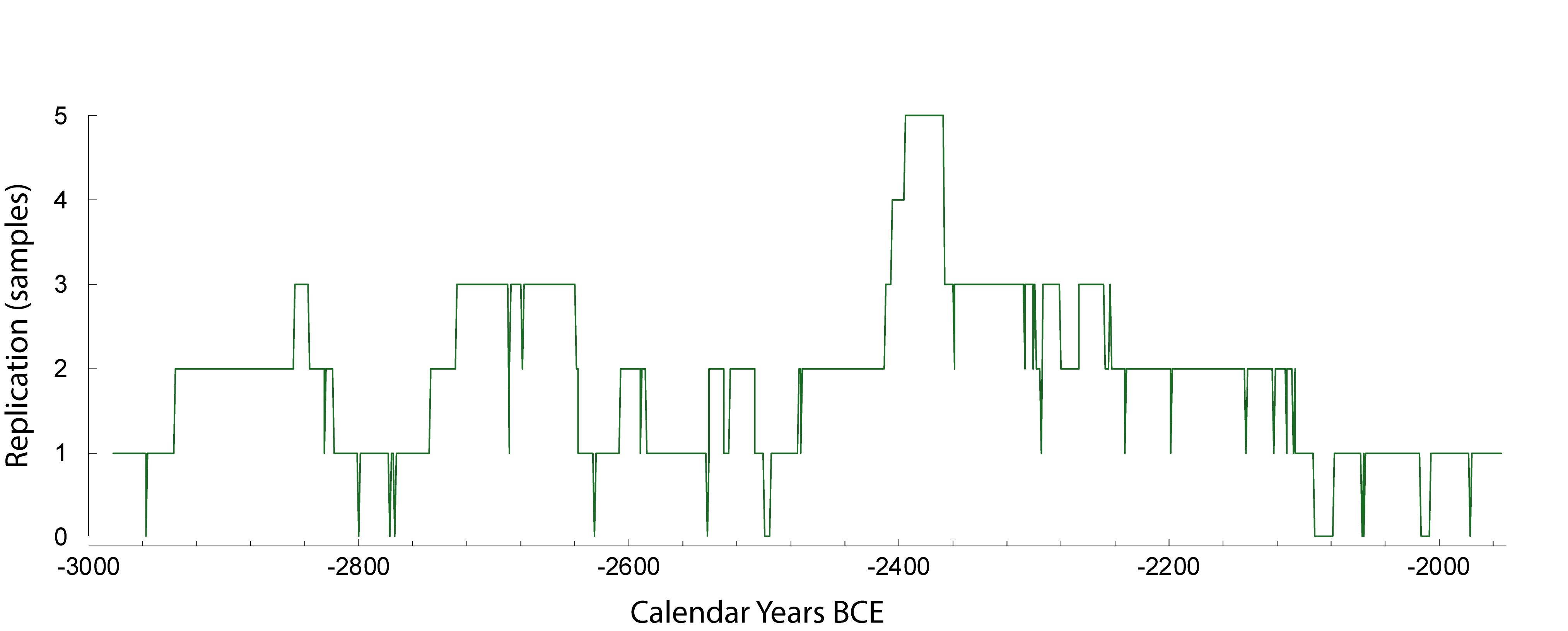


**Figure S1.** Sample replication of the reference oak stable oxygen isotope chronology used in this study and currently in development at Swansea University, UK.


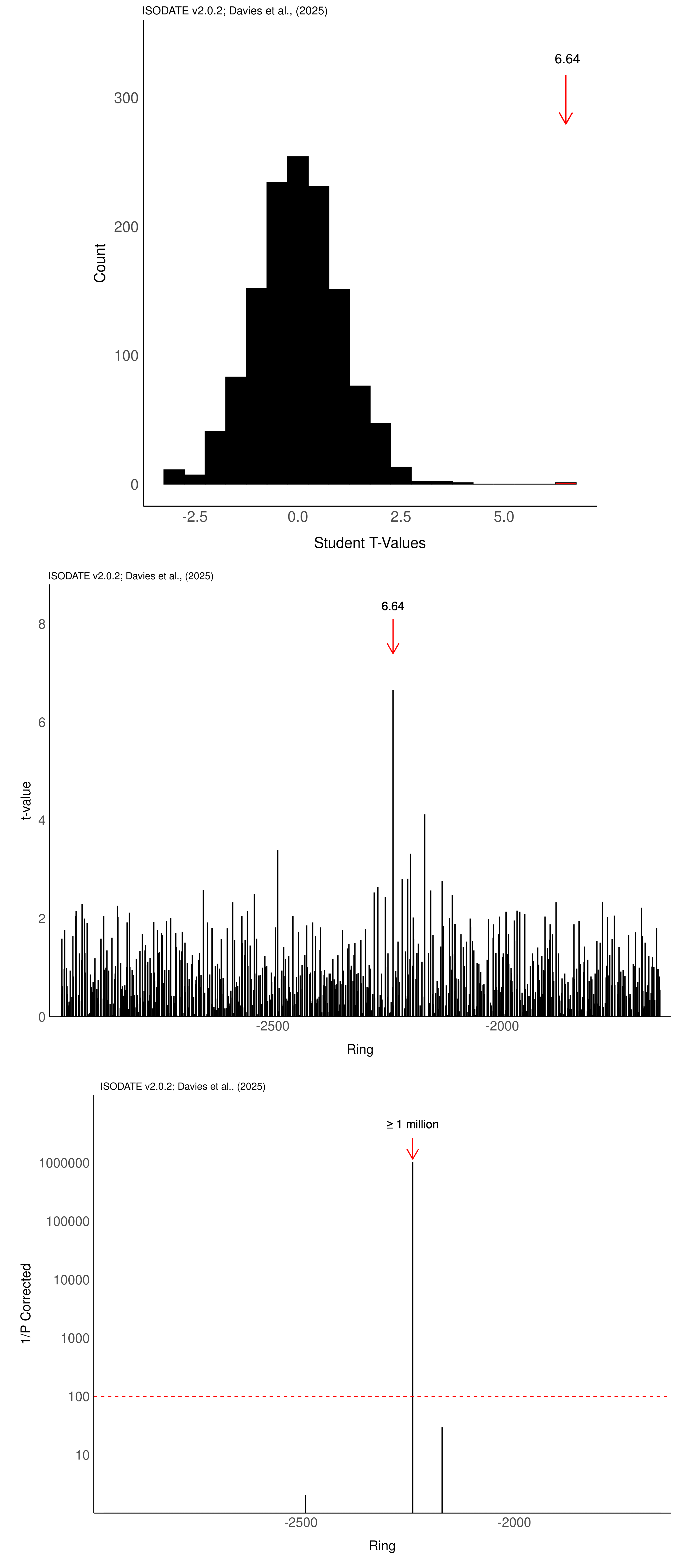


**Figure S2.** Statistical comparison of the mean of the eight cross-dated yew δ^18^O series against the oak δ^18^O reference chronology, produced using ISODATE (Davies et al., 2025).
